# Study on Quasi-Static Uniaxial Compression Properties and Constitutive Equation of Spherical Cell Porous Aluminum-Polyurethane Composites

**DOI:** 10.3390/ma11071261

**Published:** 2018-07-23

**Authors:** Haiying Bao, Aiqun Li

**Affiliations:** 1 School of Civil Engineering, Southeast University, Nanjing 210096, China; 230159406@seu.edu.cn; 2 Beijing Advanced Innovation Center for Future Urban Design, Beijing University of Civil Engineering and Architecture, Beijing 100044, China

**Keywords:** spherical cell porous aluminum (SCPA), polyurethane filler, uniaxial compression properties, relative density, constitutive equation

## Abstract

Quasi-static uniaxial compression properties and the constitutive equation of spherical cell porous aluminum-polyurethane composites (SCPA-PU composites) were investigated in this paper. The effects of relative density on the densification strain, plateau stress and energy absorption properties of the SCPA-PU composites were analyzed. It is found that the stress-strain curves of SCPA-PU composites consist of three stages: The linear elastic part, longer plastic plateau segment and densification region. The results also demonstrate that both the plateau stress and the densification strain energy of the SCPA-PU composites can be improved by increasing the relative density of the spherical cell porous aluminum (SCPA), while the densification strain of the SCPA-PU composites shows little dependence on the relative density of the SCPA. Furthermore, the applicability of three representative phenomenological models to the constitutive equations of SCPA-PU composites are verified and compared based on the experimental results. The error analysis result indicates that the Avalle model is the best model to characterize the uniaxial compression constitutive equation of SCPA-PU composites.

## 1. Introduction

Porous aluminum has gained a considerable amount of attention due to its good physical properties and excellent mechanical characteristics [1,2,3]. Moreover, it is generally accepted that porous aluminum with a spherical cell shows better structural homogeneity and mechanical performance than non-spherical cell porous aluminum [4,5]. Thus, great efforts have been made to manufacture and investigate the mechanical properties of spherical cell porous aluminum (SCPA) in recent years [6,7,8]. Open-cell SCPA can be produced by designing several small openings in different directions on the spherical cell wall. Therefore, the SCPA with high permeability incorporates substantial cell walls relative to traditional open-cell porous aluminum, wherein the cell wall is reduced to the bar-beam system. The structure of this pattern leads to the greater energy consumption, which is associated with complex failure modes of the cell membranes; furthermore, the SCPA shows superior functionality compared with closed-cell porous aluminum in certain applications. Nonetheless, it is still difficult to achieve a given energy absorption target, for example, vehicle collision energy, bridge pounding energy that suffers from strong earthquakes, simply by controlling the porosity and other structural parameters of the SCPA because of the imperfection of current fabrication approaches. Hence, it is necessary to ameliorate the issue via a simple and effective method. In fact, the attempt to enhance the mechanical performance of porous aluminum has a long history.

Alizadeh et al. [7] manufactured open-cell Al-Al_2_O_3_composite foams, using the space-holder method, and investigated the mechanical properties and energy absorption behavior of open cell Al foams, containing different volume fractions of Al_2_O_3_. Du et al. [9] examined the effect of nanoparticles on the micro-structure, compressive performance and energy absorption of Al foams. Furthermore, Sun et al. [10,11] prepared nanocopper coated aluminum foam and studied the mechanical properties of Al/Cu hybrid foam via experimental investigation and numerical modeling. Li et al. [12] reported the mechanical properties of open-cell aluminum foam wrapped with zinc film and highlighted the influence of coating time on the mechanical characteristics. The above-mentioned enhancement methods can be attributed to the addition of alloying elements and hard particles to strengthen the cell wall of aluminum foams. This idea has been utilized, and illustrated by Duarte and Ferreira [13] in detail, in many cases. However, recently, another alternative to increase the mechanical properties of porous aluminum was proposed by the introduction of polymers, owing to its simplify and effectivity. In fact, the concept of combining the advantages of porous aluminums and polymers is receiving renewed attention. Cheng and Han [14] developed a type of aluminum foam-silicate rubber composite and examined the effect of filler on the compressive behavior and energy absorption. Kitazono et al. [15] strengthened closed-cell aluminum foam using polyester resin and highlighted the impact of surface treatment methods on the compressive strength and energy absorption. Vesenjak et al. [16,17] prepared porous materials-silicone rubber composites and investigated the -influences of the base materials, specimen size and strain rate on the compressive performances and energy absorption capacity of composites. Kishimoto et al. [18] analyzed the mechanical properties of closed-cell aluminum foam-polyurethane and closed-cell aluminum foam-epoxy composites by measuring deformation distributions, adopting the digital image correlation method. Based on their studies, Yuan et al. [19] produced closed-cell aluminum foam epoxy resin composites and discussed the effect of the composite form, the relative density and the content of epoxy resin on the mechanical characteristics and energy absorption. Moreover, they presented a mathematical model to describe the plateau stress and energy absorption capacity. Furthermore, Liu et al. [20] validated the effectiveness of polyurethane (PU) for increasing the damping of open-cell aluminum foam by cyclic compression tests. Nevertheless, the aforementioned studies concerning porous aluminum-polymer composites are limited to non-spherical cell porous aluminum. PU is one of the most commonly utilized polymers in the energy absorption systems; moreover, its superior damping capacity and easy filling property have been proved [20]. Consequently, PU is adopted as the filling polymer here to improve the mechanical properties of the SCPA.

The design of the structural components applied to engineering fields is generally carried out by simulation code, based on the finite element method. The mathematical description of the mechanical behavior of the materials by a good representation of the stress-strain curve is required when performing finite element modeling and analysis. Theoretical and numerical models have recently been proposed to describe the uniaxial compression stress-strain behavior of porous materials. A micro-mechanical model related to the deformation mechanism of structure was presented by Gibson [21]. However, the micro-mechanical model was quite difficult to execute, owing to its need for a rough analysis of the porous structure. Fortunately, several phenomenological models, which aim to supply the best fitting of the experimental mechanical behavior without a direct relationship with the physics of the phenomenon, have been developed to promote the applicability of porous materials in recent years. The Rusch model [22,23,24], Liu and Subhash model [25], and Avalle model [26] are the three representative phenomenological models that characterize the stress-strain behavior of porous materials due to simple formulation and high accuracy. However, study on the constitutive model of porous aluminum-polymer composites in uniaxial compression is rarely reported. The complexity of the structure of porous aluminum-polymer composites is enhanced because of the introduction of polymer, which makes the micro-mechanical model more difficult to use. Therefore, the constitutive equation of spherical cell porous aluminum-polyurethane (SCPA-PU) composites was examined here, based on the aforementioned three phenomenological models.

SCPA-PU composites were prepared using the infiltration method, under a uniform pressure of around 0.5 MPa, in the present work. The comparison of uniaxial compression stress-strain behavior between the SCPA and SCPA-PU composites is made, and the deformation mechanism of SCPA-PU composites is analyzed. Moreover, the effects of the relative density of the SCPA on the densification strain, plateau stress and energy absorption capacity of SCPA-PU composites are investigated. Based on the experimental verification and error analysis, the most suitable model to describe the uniaxial compression stress-strain behavior of SCPA-PU composites is selected from Rusch model, Liu and Subhash model, and Avalle model, respectively.

## 2. Experimental Procedure

### 2.1. Specimen Preparations

**Figure 1:**
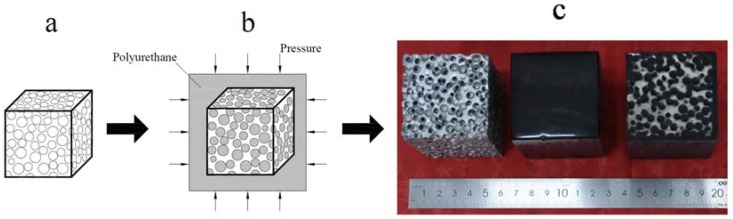
Fabrication procedure of the spherical cell porous aluminum-polyurethane composites (SCPA-PU composites): (**a**) The specimen of the spherical cell porous aluminum (SCPA); (**b**) fabrication method of the SCPA-PU composites; and (**c**) image of specimens from left to right: SCPA, polyurethane (PU), and SCPA-PU composites.

The open-cell SCPA in this paper was fabricated by the space holder method [7] and supplied by Qiangye Metal Foam Ltd (Beijing, China). The cell size is 5 mm, while the base material is Al99.7%.4-6 openings with a size of 1–1.5 mm are arranged with different orientations and situated in the cell wall of the spherical cell. Compressive samples, with the dimensions of 50 mm × 50 mm × 75 mm, were produced using a line cutting machine. The relative density value of the SCPA specimen, which is defined as the ratio of the density of the SCPA and the density of the matrix aluminum, varies from 0.263 to 0.374. At least three specimens were prepared for each material. PU is provided by Haida Rubber and Plastic Ltd. (Wuxi, China), which is usually used as an energy-absorbing material. The manufacturer’s data indicate that the density is 1.123 g/m^3^, the tensile strength is approximately 4 MPa, and the elongation at the break is 655%. The SCPA should be wrapped in PU so as to reduce the volume shrinkage of PU. The SCPA-PU composites were prepared employing the procedure shown in Figure 1. A uniform pressure of around 0.5 MPa was applied to press the PU elastomer into the SCPA. Finally, the specimens of the SCPA-PU composites were produced after they were heated at 100 °C for ten hours. The open-cell spherical cell could be filled with PU due to the excellent fluidity and longer curing time of PU. Three kinds of specimens, which are named SCPA, PU and SCPA-PU composites, are illustrated in Figure 1c.

### 2.2. Compressive Test

**Figure 2:**
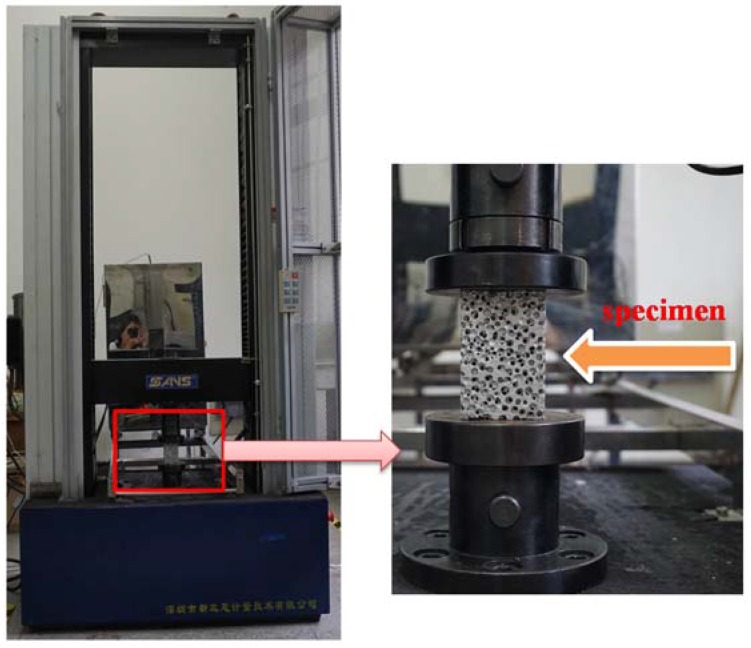
Uniaxial compression tests utilizing CMT5105 electron universal testing machine.

Quasi-static uniaxial compressive tests were performed using a CMT5105 electron universal testing machine (SANS, Minneapolis, MN, USA) (shown in Figure 2) at room temperature (23 °C) under displacement control at a constant cross-head speed of 4.5 mm/min. The circular aligned platens were coated with silicon greases to reduce surface friction with the compression specimens. The variations of load and displacement were automatically recorded by the machine. It is worth noting that the experimental results shown in this paper correspond to the average of multiple specimens tested.

## 3. Results and Discussion

### 3.1. Compressive Stress-Strain Behavior

**Figure 3:**
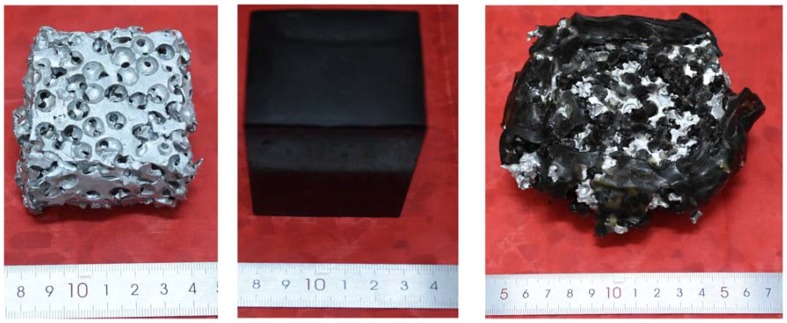
Deformed SCPA, PU and SCPA-PU composites specimens after uniaxial compression tests.

**Figure 4:**
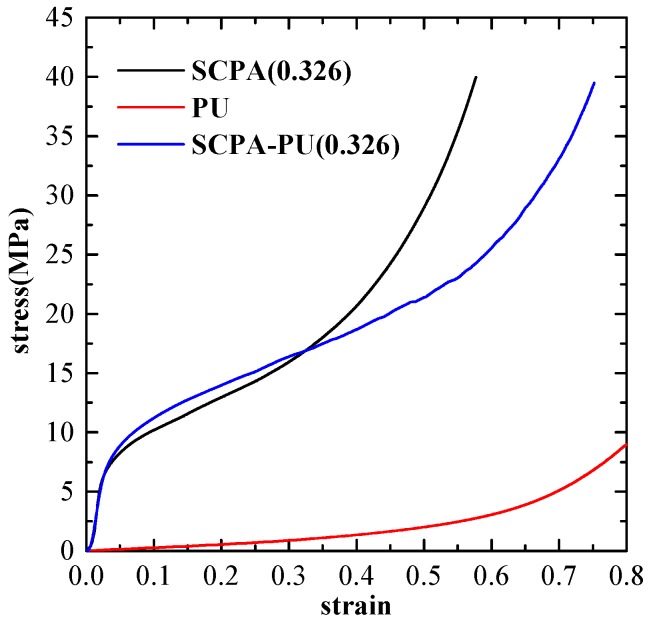
Compressive stress-strain curves of SCPA, PU and SCPA-PU composites.

The appearance of three types of deformed specimens after the uniaxial compression test is shown in Figure 3, indicating their different deformation modes. The comparison of the compressive stress-strain curves between SCPA, PU, and SCPA-PU composite specimens is made in Figure 4. The stress of PU increases as the strain increases without yielding, which is the typical behavior of elastomer. The stress-strain curves of the SCPA-PU composites appeared to have three similar regions of unfilled SCPA [1], i.e., the linear elastic part, the plastic plateau segment and the densification regions. However, compared with the stress-strain curve of SCPA, the compressive stress-strain curve of the SCPA-PU composites exhibits a longer and higher plateau region.

The main deformation mechanism of SCPA, which is described by the homogeneous failure mode with multiple random deformation bands, is reported [8]. Nevertheless, the deformation mechanism of SCPA-PU composites is quite different from that of SCPA, which is ascribed to the introduction of PU. The result presented in Figure 4 shows that the stress is mainly borne by the cell wall of the SCPA because of its greater strength over PU at the stage of low strain level, thus, the compressive curve of the SCPA-PU composites coincides with that of SCPA at the early stage of deformation. Resistance to deformation of the cell wall increases as the compressive stress increases, which is related to the PU filling the spherical cell. Meanwhile, the lateral deformation of the SCPA-PU composites becomes larger with the increase of load owing to the incompressibility of PU volume, which is demonstrated by the images of PU and SCPA-PU composite specimens after compression shown in Figure 3. The lateral deformation is restrained by the cell wall, which, conversely, raises the resistance of the SCPA-PU composites. Moreover, the incompressibility of PU postpones the yield and buckling of the cell edges, which makes the plastic deformation capacity of the SCPA-PU composites stronger than that of the SCPA. Moreover, this conclusion is consistent with that in the quasi-static uniaxial compression of open-cell aluminum foam with silicate rubber [14].

### 3.2. Energy Absorption Characteristics

**Figure 5:**
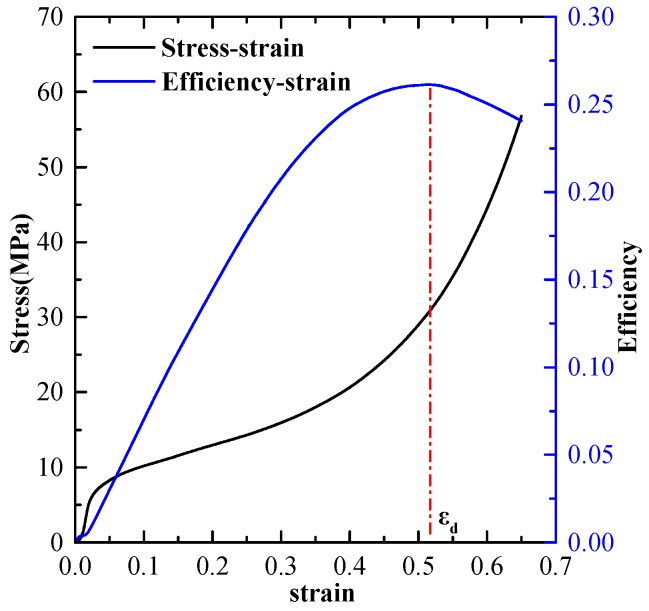
Calculated uniaxial compressive stress-strain curve and efficiency-strain curve.

One of the important mechanical properties for the evaluation of the application of porous materials is the energy absorption characteristic. It is widely accepted that both the densification strain and the plateau stress play important roles in characterizing the energy absorption capacity of porous materials. Where the densification strain is determined using the energy absorption efficiency, potential mistakes caused by the existing huge uncertainties in other methods can be avoided [27]. The optimal energy absorption of porous materials can be identified by an energy efficiency parameter η(ε):

(1) $$\eta \left(\varepsilon \right)=\frac{1}{\sigma \left(\varepsilon \right)}\int_{\varepsilon_{y}}^{\varepsilon }\sigma \left(\varepsilon \right)d\varepsilon ,$$

where $\varepsilon_{y}$ is the strain corresponding to the starting point of the plateau segment. Furthermore, a representative strain of densification, $\varepsilon_{d}$, is determined as:

(2) $${\frac{d\eta \left(\varepsilon \right)}{d\varepsilon }|}_{\varepsilon =\varepsilon_{d}}=0,$$

at which the energy absorption efficiency, η(ε), reaches a maximum value on the efficiency-strain curve, i.e., the tangential stiffness is equal to zero, as shown in Figure 5. The plateau stress, $\sigma_{pl}$, is expressed as:

(3) $$\sigma_{pl}=\frac{\int_{\varepsilon_{y}}^{\varepsilon_{d}}\sigma \left(\varepsilon \right)d\varepsilon }{\varepsilon_{d}-\varepsilon_{y}},$$

as $\varepsilon_{y}$ is usually very small compared with $\varepsilon_{d}$, it is assumed to be zero here. The densification strain energy, $W_{\varepsilon_{d}}$, which is a significant index for the characterization of the energy absorption capacity of porous materials, is defined as:

(4) $$W_{\varepsilon_{d}}=\int_{\varepsilon_{y}}^{\varepsilon_{d}}\sigma \left(\varepsilon \right)d\varepsilon ,$$

The above presented methods are utilized to calculate the densification strain, the plateau stress and the densification strain energy here, respectively.

**Figure 6:**
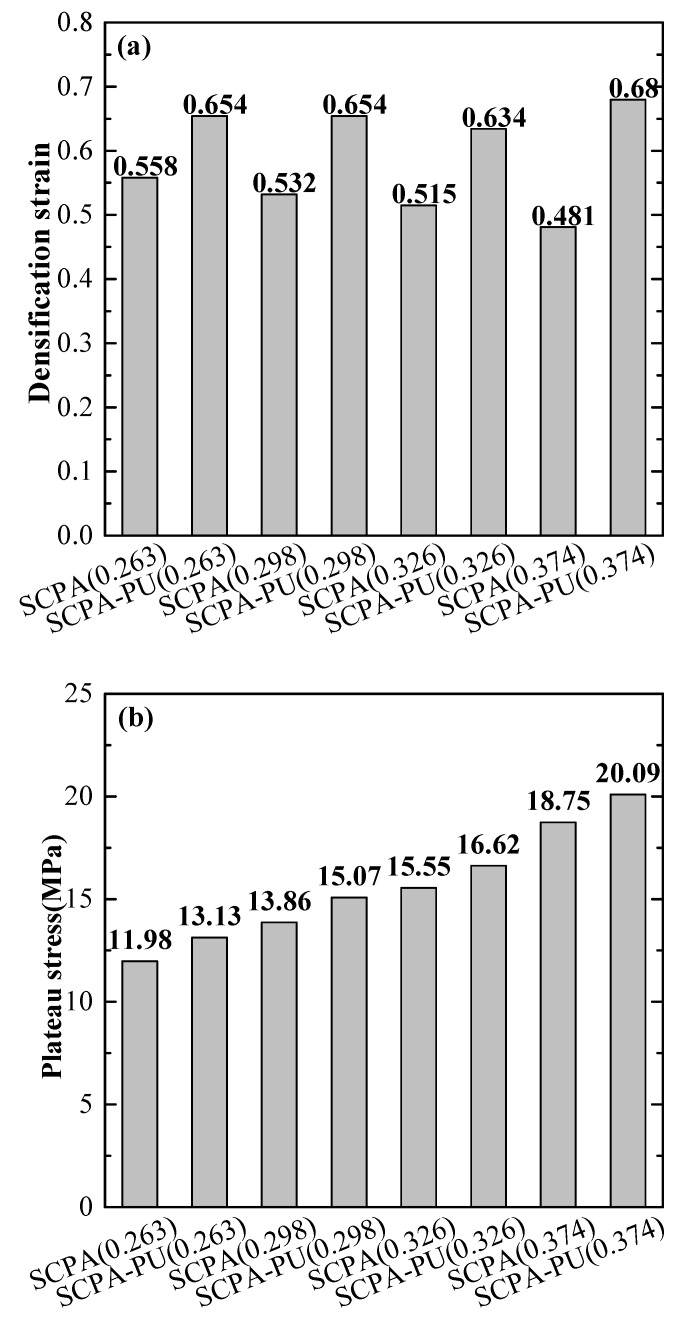

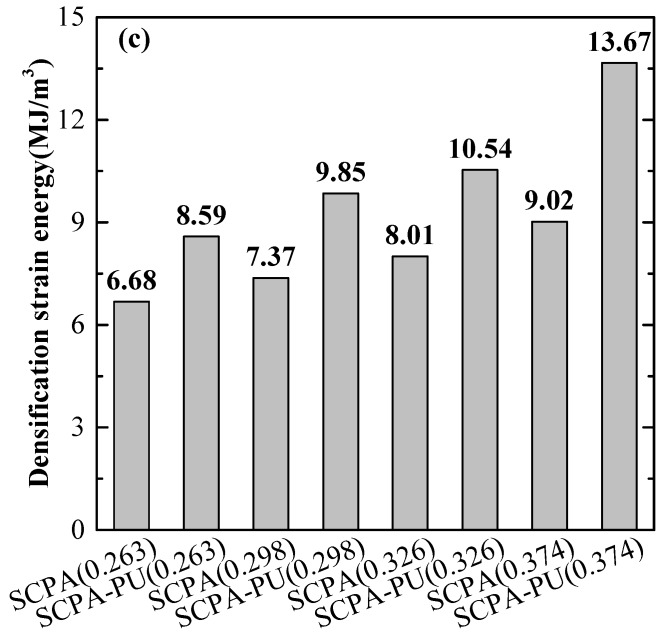
Comparison of SCPA and SCPA-PU composites specimens, with different relative density values for (**a**) the densification strain; (**b**) plateau stress; and (**c**) densification strain energy.

The densification strain, plateau stress and densification strain energy of the SCPA and the SCPA-PU composites, with different relative densities, are shown in Figure 6a–c, respectively. The impact of PU is more pronounced on the densification strain and densification strain energy of the SCPA, as compared with the plateau stress, as shown in Figure 6, which is associated with the low strength and high elasticity of the PU. The densification strain values of the SCPA-PU composites, with the relative density values of 0.263, 0.298, 0.326 and 0.374, are 17.2%, 22.93%, 23.11% and 33.06% higher than those of the SCPA, respectively. At the same time, the plateau stress values of the SCPA-PU composites, with the relative density values of 0.263, 0.298, 0.326 and 0.374, are 9.6%, 8.73%, 6.88% and 7.15% higher than those of the SCPA, respectively. Moreover, the densification strain energy values of the SCPA-PU composites, with the relative density values of 0.263, 0.298, 0.326 and 0.374, are 28.59%, 33.65%, 31.59% and 51.55% higher than those of the SCPA, respectively.

The dependence of the densification strain value of the SCPA-PU composites on the relative density is drastically reduced (Figure 6a) when compared with the plateau stress value (Figure 6b) and densification strain energy value (Figure 6c). The densification strain value of the SCPA-PU composites, studied in this paper, can be controlled at about 0.65. Furthermore, it is seen from Figure 6b,c that the relationship between the plateau stress of the SCPA-PU composites and the relative density is similar to that of the densification strain energy and relative density. The two indexes increase with the relative density, and the weak dependence of the densification strain on the relative density is caused by this coupling effect.

In summary, the energy absorption capacity of the SCPA is enhanced by the introduction of the PU. Moreover, the plateau stress value and densification strain energy value of the SCPA-PU composites increase as the relative density value increases, while the relationship between the densification strain and the relative density of the SCPA-PU composites is relatively insignificant.

The ideal energy absorption efficiency, which was proposed by Miltz and Gad [28] to assess whether the porous material is an idealized energy absorption material, is introduced here to further evaluate the energy absorption property of the SCPA-PU composites, which is formulated as:

(5) $$I=\frac{\int_{0}^{\varepsilon_{m}}\sigma \left(\varepsilon \right)d\varepsilon }{\sigma_{m}\varepsilon_{m}},$$

where $\sigma_{m}$ is the stress associated with the strain, $\varepsilon_{m}$, and the bigger the I value, the closer the porous material is to the ideal energy absorption material.

**Figure 7:**
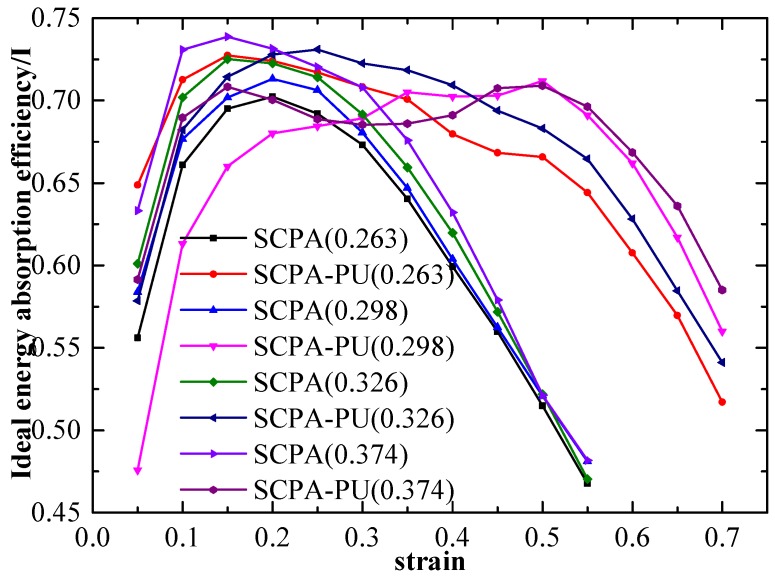
I−ε curves of the SCPA and the SCPA-PU composites with different relative density values.

Comparisons of the I value for the SCPA and the SCPA-PU composites, with different relative density values, are made in Figure 7. It can be seen from Figure 7 that the I consists of three stages in all cases: Fast ascending branch (I), where the I increases monotonously to a high energy absorption efficiency point with the compressive strain; plateau stage (II), in which the I maintains the high efficiency level, but some fluctuation exits as the strain increases; and descending region (III), where the I decreases with the increase of the compressive strain. Furthermore, as shown in Figure 7, the average I value of the SCPA, with the relative density value of 0.263, is 0.685 when the strain is between 0.1 and 0.30, while the average I value of the SCPA-PU composites, with the relative density value of 0.263, is 0.7 when the strain is between 0.1 and 0.5. Meanwhile, the average I value of the SCPA, with the relative density value of 0.298, is 0.696 when the strain is between 0.1 and 0.30, while the average I value of the SCPA-PU composites, with the relative density value of 0.298, is 0.692 when the strain is between 0.15 and 0.50. Moreover, the average I value of the SCPA, with the relative density value of 0.326, is 0.711 when the strain is between 0.1 and 0.3, while the average I value of the SCPA-PU composites, with the relative density value of 0.326, is 0.705 when the strain is between 0.1 and 0.55. Besides, the average I value of the SCPA, with the relative density value of 0.374, is 0.726 when the strain is between 0.1 and 0.3, while the average I value of the SCPA-PU composites, with the relative density value of 0.374, is 0.696 when the strain is between 0.1 and 0.55.

Based on the above experimental results, the following two conclusions can be drawn: (1) The plateau I values of the SCPA-PU composites is close to those of the SCPA, however, a wider plateau strain range of the SCPA-PU composites is presented when compared with the plateau strain range of the SCPA; and (2) the I value, situated in the plateau stage on the relative density of the SCPA-PU composites, has a weak dependence.

### 3.3. Uniaxial Compression Constitutive Equation of SCPA-PU Composites

Among the existing phenomenological models regarding the constitutive equation of porous materials, the Rusch model [22,23,24], Liu and Subhash model [25] and Avalle model [26] are the most commonly employed. The three models are adopted and fitted to the experimental results of the SCPA-PU composites here, and the best-fitting model for describing the SCPA-PU composites among these three is quantitatively identified in terms of the metric of root mean square error.

#### 3.3.1. Existing Phenomenological Models

##### Rusch Model

The Rusch model is a phenomenological model with a simple expression, which is presented by the sum of two power functions:

(6) $$\sigma =a\varepsilon^{p}+b\varepsilon^{q},0<p<1,q>1,$$

where σ and ε are the nominal stress and strain, respectively, and a,b,p,q are empirically determined. The first term is designed for the elastic-plateau region, while the second term is utilized for modelling the densification region. Generally, the inaccuracy in describing the densification phase of porous materials is a drawback of the model when, as a consequence of compression, the internal voids gradually disappear. 

##### Liu and Subhash Model

The model proposed by Liu and Subhash similarly consists of two parts, the first describing the elastic-plastic stage and the second one representing the densification segment, and is shown as follows:

(7) $$\sigma =A\frac{e^{\alpha \varepsilon }-1}{B+e^{\beta \varepsilon }}+e^{C}\left(e^{\gamma \varepsilon }-1\right),$$

the function has six parameters, wherein the parameter *A* is related to the yield stress, *B* plays a role in shifting the lower asymptote, the behavior of the plateau region is determined by the difference between α and β, the parameter *C* plays a role in stretching or shrinking of the curve, and the speed of the densification is controlled by the γ. The fundamental compressive and tensile stress-strain behavior of porous materials with various initial densities under large deformation can be captured by the model. Moreover, the equation is continuously differentiable.

##### Avalle Model

Recently, a model, which is composed of the elastic-plastic part and the densification segment, was given by Avalle as follows:

(8) $$\sigma =F\left(1-e^{-\left(G/F\right)\varepsilon {\left(1-\varepsilon \right)}^{m}}\right)+H{\left(\frac{\varepsilon }{1-\varepsilon }\right)}^{n},$$

the parameters of the model can be empirically determined, wherein the plateau stress is defined by the parameter *F*, the parameter *G* is adopted to represent the initial elastic modulus, the curve knee at the connection of the elastic stage with the plateau region is achieved by the appropriate choice of the parameter m, the curve change trend of the densification process of porous materials is affected by the parameter n. The second term of the model is a modification of the second one of the Rusch model and has been introduced to obtain a vertical asymptote corresponding to the physical limit of compression (ε=1).

#### 3.3.2. Evaluation of Model Performance

**Figure 8:**
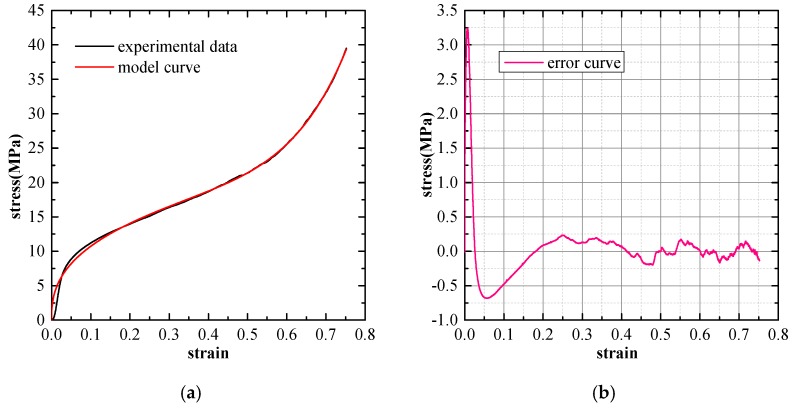
(**a**) Comparison between the curve predicted by the Rusch model and the experimental curve ($\rho^{*}/\rho^{s}$ = 0.326); and (**b**) model prediction error.

**Figure 9:**
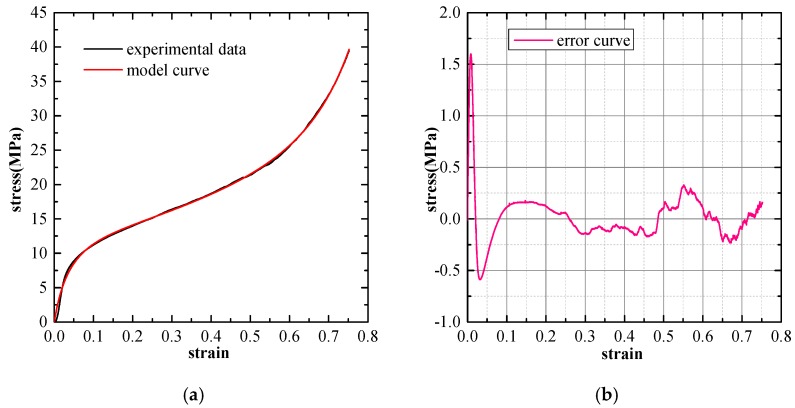
(**a**) Comparison between the curve predicted by the Liu and Subhash model and the experimental curve ($\rho^{*}/\rho^{s}$ = 0.326); and (**b**) model prediction error.

**Figure 10:**
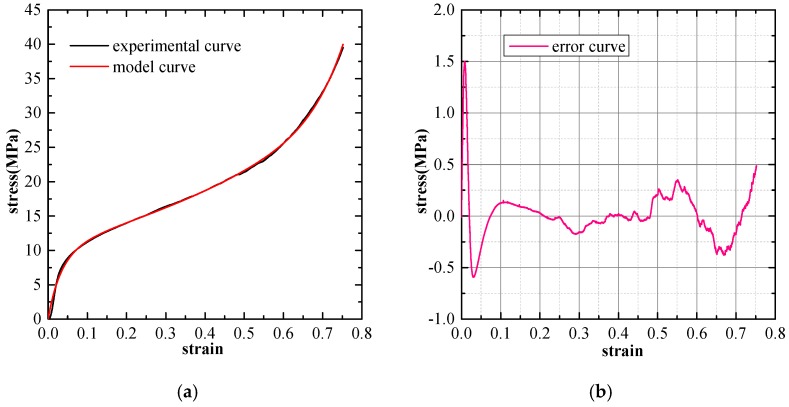
(**a**) Comparison between the curve predicted by the Avalle model and the experimental curve ($\rho^{*}/\rho^{s}$ = 0.326); and (**b**) model prediction error.

The Experimental verification and error analysis of the three mentioned types of phenomenological models are performed based on the stress-strain curves of SCPA-PU composites. The least square method is adopted here to compute the deviation of the model prediction value from the experimental results. Consequently, the model prediction error is taken as the difference between the experimental stress and the model stress at the same strain value. The curve fitting the results of the three considered models for the SCPA-PU composites with the relative density value of 0.326 are separately shown in Figure 8, Figure 9 and Figure 10. The prediction error of each model is shown in the right diagram of each figure, which is expressed as a function of the strain. It is obviously seen that the fitting performance of the three models is the worst in the elastic region, while a certain level of improvement is observed in the plateau and densification region. Furthermore, the fitting results also indicate that the three selected models can be employed to characterize the uniaxial compression stress-strain behavior of the SCPA-PU composites. Additionally, model forecast error results show that the fluctuation of the error curve for the Rusch model is the most significant, while the quality of the fitting between Avalle model and Liu and Subhash model are superior to the Rusch model and appear to be comparable.

**Figure 11:**
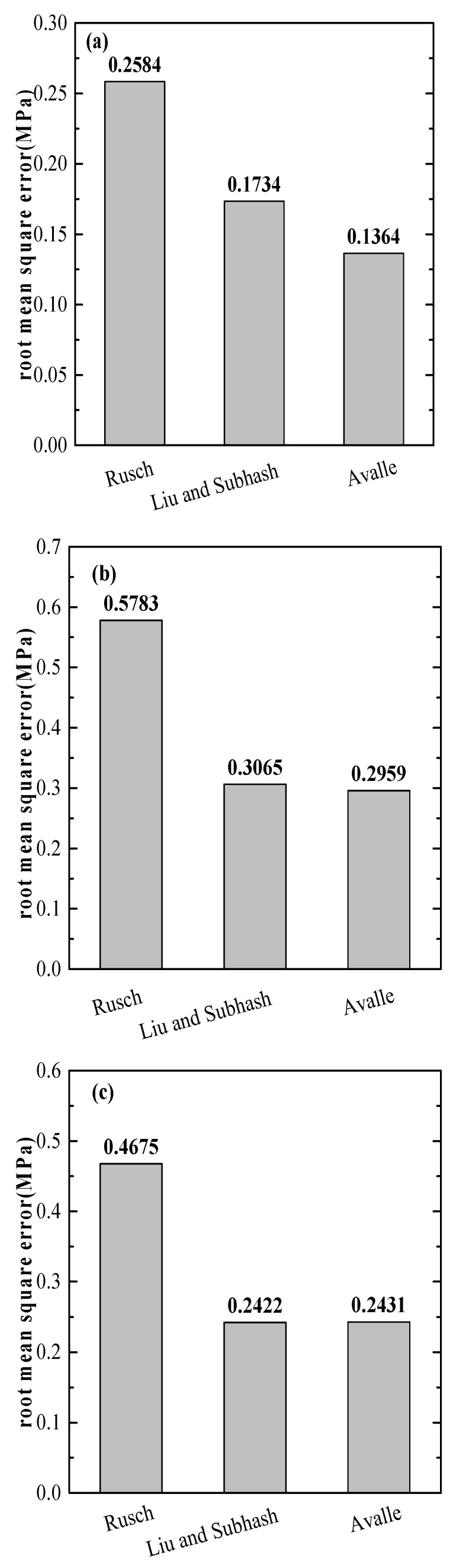

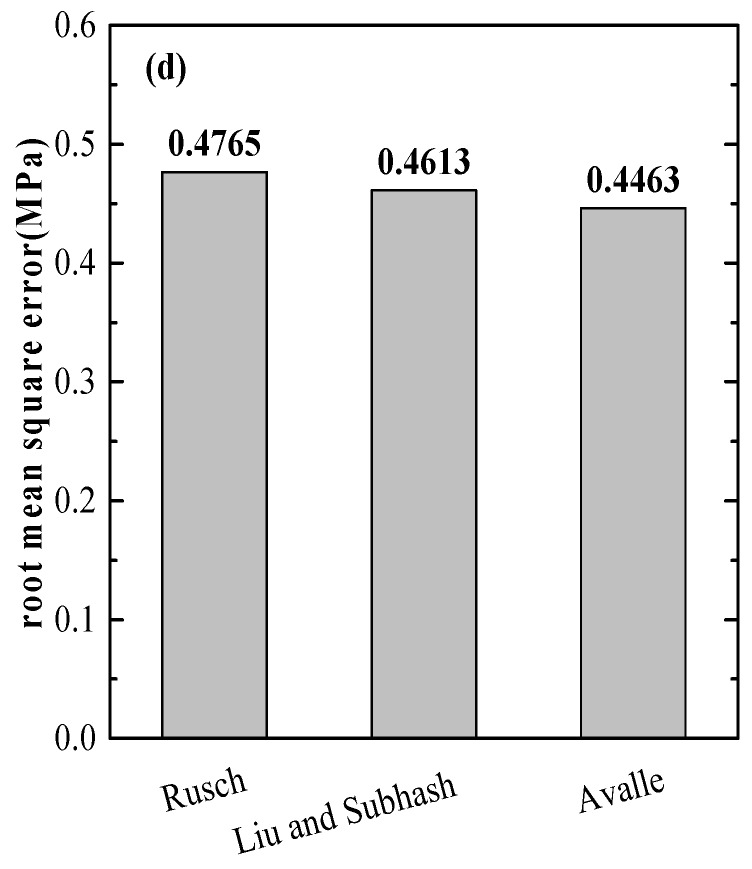
Comparison of the root mean square error of each model for (**a**) 0.263; (**b**) 0.298; (**c**) 0.326; and (**d**) 0.374 relative density values.

In order to choose the most suitable model from the three mentioned phenomenological models for SCPA-PU composites, a direct comparison of the overall fitting ability of the three considered models can be conducted by means of the root mean square error (RMSE) for SCPA-PU composites with different relative density values: They are presented with histograms in Figure 11. It is found that the worst fitting behavior of the Rusch model is displayed regardless of the relative density value, while the excellent fitting performance of the Avalle model is seen among the chosen relative density value. It can be concluded that the Avalle model is the best phenomenological model to characterize the uniaxial compression constitutive equation of SCPA-PU composites among the three selected models.

## 4. Conclusions

1. The compressive stress-strain curves of spherical cell porous aluminum-polyurethane composites (SCPA-PU composites) consist of three stages: Linear elastic part, plateau region and densification segment. Furthermore, PU is beneficial to the increase of the plateau stress and elongation of the densification strain.
2. The energy absorption capacity of SCPA-PU composites is superior to that of the SCPA. The densification strain energy of the SCPA-PU composites, with the relative density values of 0.263, 0.298, 0.326, and 0.374, is 28.59%, 33.65%, 31.59%, and 51.55% higher than those of the SCPA, with the same relative density value, respectively. Besides, the weak dependence of the densification strain of the SCPA-PU composites on the relative density is seen, while the plateau stress and the densification strain energy increase as the relative density increases. Furthermore, the ideal energy absorption efficiency (I)-strain curves of SCPA-PU composites and SCPA consist of three parts: Fast ascending branch, plateau stage, and descending region. The plateau I value of SCPA-PU composites is close to that of SCPA, while it has a wider plateau strain range. It is also found that the plateau I value of SCPA-PU composites is insensitive to the relative density of the SCPA.
3. Based on the calculated root mean square error results of SCPA-PU composites with different relative density values, the best phenomenological model to characterize the constitutive equation of SCPA-PU composites is the Avalle model. This conclusion provides a foundation for the following research regarding the constitutive model of SCPA-PU composites considering strain rate and temperate factors.

## References

[1] Banhart J. (2001). Manufacture, characterisation and application of cellular metals and metal foams. Prog. Mater. Sci..

[2] Lefebvre L.-P., Banhart J., Dunand D.C. (2008). Porous metals and metallic foams: Current status and recent developments. Adv. Eng. Mater..

[3] García-Moreno F. (2016). Commercial applications of metal foams: Their properties and production. Materials.

[4] Jiang B., Zhao N.Q., Shi C.S., Li J.J. (2005). Processing of open cell aluminum foams with tailored porous morphology. Scr. Mater..

[5] Su Y., Li Z., Gong X., Ouyang Q., Guo Q., Guo C., Zhang J., Zhang D. (2016). Structural modeling and mechanical behavior of Metal-Porous-Polymer-Composites (MPPCs) with different polymer volume fractions. Compos. Struct..

[6] Jiang B., Wang Z., Zhao N. (2007). Effect of pore size and relative density on the mechanical properties of open cell aluminum foams. Scr. Mater..

[7] Alizadeh M., Mirzaei-Aliabadi M. (2012). Compressive properties and energy absorption behavior of Al–Al_2_O_3_ composite foam synthesized by space-holder technique. Mater. Des..

[8] Fan Z., Zhang B., Gao Y., Guan X., Xu P. (2018). Deformation mechanisms of spherical cell porous aluminum under quasi-static compression. Scr. Mater..

[9] Du Y., Li A.B., Zhang X.X., Tan Z.B., Su R.Z., Pu F., Geng L. (2015). Enhancement of the mechanical strength of aluminum foams by SiC nanoparticles. Mater. Lett..

[10] Sun Y., Burgueño R., Vanderklok A.J., Tekalur S.A., Wang W., Lee I. (2014). Compressive behavior of aluminum/copper hybrid foams under high strain rate loading. Mater. Sci. Eng. A.

[11] Sun Y., Burgueño R., Wang W., Lee I. (2015). Modeling and simulation of the quasi-static compressive behavior of Al/Cu hybrid open-cell foams. Int. J. Solids Struct..

[12] Li Z., Huang Y., Wang X., Wang X., Wang D., Han F. (2016). Enhancement of open cell aluminum foams through thermal evaporating Zn film. Mater. Lett..

[13] Duarte I., Ferreira J.M.F. (2016). Composite and nanocomposite metal foams. Materials.

[14] Cheng H.F., Han F.S. (2003). Compressive behavior and energy absorbing characteristic of open cell aluminum foam filled with silicate rubber. Scr. Mater..

[15] Kitazono K., Suzuki R., Inui Y. (2009). Novel strengthening method of closed-cell aluminum foams through surface treatment by resin. J. Mater. Process. Technol..

[16] Vesenjak M., Krstulović-Opara L., Ren Z., Öchsner A., Domazet Ž. (2009). Experimental study of open-cell cellular structures with elastic filler material. Exp. Mech..

[17] Vesenjak M., Krstulović-Opara L., Ren Z. (2013). Characterization of irregular open-cell cellular structure with silicone pore filler. Polym. Test..

[18] Kishimoto S., Wang Q., Tanaka Y., Kagawa Y. (2014). Compressive mechanical properties of closed-cell aluminum foam–polymer composites. Compos. Part B.

[19] Yuan J., Chen X., Zhou W., Li Y. (2015). Study on quasi-static compressive properties of aluminum foam-epoxy resin composite structures. Compos. Part B.

[20] Liu S., Li A., He S., Xuan P. (2015). Cyclic compression behavior and energy dissipation of aluminum foam–polyurethane interpenetrating phase composites. Compos. Part A.

[21] Gibson L.J., Ashby M.F. (1999). Cellular Solids: Structure and Properties.

[22] Rusch K.C. (1969). Load–compression behavior of flexible foams. J. Appl. Polym. Sci..

[23] Rusch K.C. (1970). Load-compression behavior of brittle foams. J. Appl. Polym. Sci..

[24] Rusch K.C. (1970). Energy-absorbing characteristics of foamed polymers. J. Appl. Polym. Sci..

[25] Liu Q., Subhash G. (2004). A phenomenological constitutive model for foams under large deformations. Polym. Eng. Sci..

[26] Avalle M., Belingardi G., Ibba A. (2007). Mechanical models of cellular solids: Parameters identification from experimental tests. Int. J. Impact Eng..

[27] Li Q.M., Magkiriadis I. (2006). Harrigan, J.J. Compressive strain at the onset of densification of cellular solids. J. Cell. Plast..

[28] Miltz J., Gruenbaum G. (1981). Evaluation of cushioning properties of plastic foams from compressive measurements. Polym. Eng. Sci..

